# Implementation of a Medical Education Rotation for Senior Emergency Medicine Residents

**DOI:** 10.21980/J8BH17

**Published:** 2021-07-15

**Authors:** Deena Ibrahim Bengiamin, Lizveth Fierro, Molly Estes, Michael Kiemeney, Timothy Patrick Young

**Affiliations:** * Laboratory for Innovations in Medical Education, Loma Linda University School of Medicine, Department of Emergency Medicine, Loma Linda University Medical Center, Loma Linda, CA

## Abstract

**Audience and type of curriculum:**

This medical education (MedEd) rotation is designed for post graduate year 3 (PGY-3) residents.

**Length of curriculum:**

The rotation runs over one month for each PGY-3.

**Introduction:**

Resident physicians have teaching responsibilities during and after training. These responsibilities expand beyond teaching medical students and junior residents to include teaching advanced practice providers, nursing colleagues, and prehospital personnel.^1^ The need for formal teaching curricula in graduate medical education is recognized, but practical examples are lacking.^2^

**Educational Goals:**

Our objectives were to provide our senior residents with exposure to various aspects of the field of MedEd, to further develop their teaching skills and to encourage them to consider a career in academic emergency medicine.

**Educational Methods:**

The educational strategies used in this curriculum include*:* 1) clinical shifts supervising small groups of medical students with dedicated faculty supervision, 2) a structured simulation-based medical student teaching activity where the resident is able to provide feedback and teach medical students, 3) a MedEd project, 4) required readings that cover a variety of topics including education theory, curriculum design, and feedback, 5) case-based didactic presentation at our monthly case conference, and 6) one hour of postgraduate year 1 (PGY-1) small group facilitation focusing on fundamentals of emergency medicine.

**Research Methods:**

PGY-3 residents completed an online survey prior to residency graduation. The timing of the survey was purposefully delayed to the end of the academic year to allow the residents time to practice techniques they learned during their MedEd rotation.

**Results:**

Thirteen residents (93%) completed a survey. Five residents (38%) reported that the rotation had “some” or more impact on their career decision. The other 8 residents reported “almost no impact” or “a little bit of impact.” Ten residents (77%) reported that they would “sometimes,” “often,” or “almost always” use the teaching techniques they learned during the rotation. The highest rated activities were simulation-based teaching and dedicated clinical teaching shifts. Confidence with bedside teaching improved after the session, with a median confidence before the session of 3/5 (moderately confident; IQR 2–3) and a median confidence after the session of 4/5 (quite confident; IQR 3–4, p=0.006).

**Discussion:**

Our MedEd rotation improved teaching confidence but had low impact on career decision. Residents rated the interactive, faculty-supervised components of the rotation highest. We recommend that programs interested in instituting a MedEd rotation first trial the rotation as an elective and utilize established formal teaching activities.

**Topics:**

Medical Education, resident physician, medical student teaching, simulation, academic medicine.

## USER GUIDE

**Table t1-jetem-6-3-c1:** 

List of Resources:	
Abstract	1
User Guide	3
Didactics and Hands on Curriculum Chart	7
Appendix A: Calendar	8


[Table t1-jetem-6-3-c1]

**Learner Audience:**

Senior Residents

**Length of Curriculum:**

1 month

**Topics:**

Medical Education, resident physician, medical student teaching, simulation, academic medicine.

**Objectives:**

By the end of this curriculum, learners will be able to:

1. Identify learners by level of training and progression of skills and knowledge
2. Learn and refine bedside teaching techniques
3. Facilitate small group learning sessions
4. Lead simulation-based learning sessions
5. Produce a high-quality medical education product
6. Critique large group lecture
7. Develop skill in learner evaluation and feedback delivery

### Brief introduction

Resident physicians regularly teach junior residents, medical students, and other hospital staff. As the number of emergency medicine (EM) teaching programs grows to include more community-based programs, more newly-graduated residents accept positions with explicitly-defined teaching responsibilities. With the growing use of advanced practice providers in EM, even graduates working in “non-teaching” institutions supervise and educate other providers.^1^ Teaching residents how to teach impacts the training of learners they encounter and better prepares them for a career in EM. Yet residents are seldom exposed to a formal teaching curriculum; more commonly, they gain teaching skills by watching mentors. The need for formal teaching curricula in graduate medical education is recognized, but practical examples are lacking.^2^

### Problem identification, general and targeted needs assessment

Residents’ perception of the teaching they receive correlates better with teacher skill and ability to establish a positive learning environment than time available for teaching.^3^ Our residents, however, have anecdotally commented that preoccupation with learning to manage a busy emergency department detracts from their ability to develop such skills. This problem and the gap between our current and ideal approach were identified formally through resident evaluations and discussions at our program evaluation committee meetings. Teaching skills may seem to develop naturally with clinical experience. However, residents have rated teaching from less experienced faculty more highly.^4^ Conversely, willingness to teach is strongly related to positive faculty teaching evaluations.^3^ A dedicated block of medical education time could improve teaching confidence and increase willingness to teach. Our Graduate Medical Education office has placed emphasis on increasing resident exposure to academia as well as their retention as faculty. To give our residents focused training and experience in medical education, we implemented a one-month Medical Education (MedEd) rotation.

### Goals of the curriculum

The objectives were to provide our senior residents with exposure to various aspects of the field of MedEd, to further develop their teaching skills, and to encourage them to consider a career in academic emergency medicine.

### Objectives of the curriculum

By the end of this curriculum, learners will be able to:

1. Identify learners by level of training and progression of skills and knowledge
2. Learn and refine bedside teaching techniques
3. Facilitate small group learning sessions
4. Lead simulation-based learning sessions
5. Produce a high-quality medical education product
6. Critique large group lecture
7. Develop skill in learner evaluation and feedback delivery

### Educational Strategies

Attached as a separate document.

### Results and tips for successful implementation: Implementation

Kern and Thomas’ 6-step approach to curriculum development for MedEd was our guiding framework.^5^ Problem identification and needs assessment occurred at our annual program evaluation committee meeting through discussion with faculty and residents. The largest barrier to implementation of a required rotation was the overall shift reduction needed to implement the rotation. At the time of implementation, resident class size was 13. Given the inability to reduce shifts for all of our senior residents, we implemented the curriculum as an elective. This also gave us an engaged smaller group of residents to trial the month and allowed us to receive and incorporate feedback. Four senior residents chose to pursue the

MedEd elective. The elective featured 4 main components: 1) clinical teaching shifts supervising small groups of medical students with dedicated faculty support,^6^ 2) a structured simulation-based medical student teaching activity called the Acute Care Skills Evaluation (ACSE) in which medical students manage decompensating patients and receive feedback and teaching related to resuscitation and patient handoffs,^7^ 3) a MedEd project, and 4) required readings that cover a variety of topics including education theory, curriculum design, and feedback. Both the ACSE and small groups shifts were pre-existing teaching activities in our department.^6^,^7^ For the ACSE, residents worked with 2 faculty members with simulation experience. They were given background reading related to the Good Judgement simulation debriefing technique^8^ and the I-PASS technique for handoffs.^9^ The ACSE provided the resident with an opportunity to lead a simulation-based session that focused on resuscitation of an acutely decompensating patient as well as provide individualized feedback to medical students. Residents received feedback on their teaching from ACSE faculty during and after the session. For the teaching shifts, residents directly supervised 4 medical students caring for real ED patients with active oversight from faculty. Neither the resident nor the faculty member was part of the regular ED shift schedule, and patients were not cared for by additional providers on that schedule. The resident functioned as a junior faculty member, listening to medical student patient presentations, directly supervising history and physical examinations, teaching at the patient’s bedside, providing individualized feedback to each student, and teaching medical students in a small group setting. The resident received teaching feedback from the faculty member during and after each session. The small group shifts and ACSE provided the resident with an opportunity to develop rapport with students as well as provide a more thorough and thoughtful evaluation over the course of several weeks rather than one clinical shift.

At the end of the first year, we again reviewed the feasibility of a required rotation. Elective feedback from our 4 participants was presented to our program evaluation committee as part of our annual program evaluation. Support for a required rotation grew among our residents and faculty as a result of feedback from elective participants. Our resources for implementation grew as well as a result of an increase in complement. During the first year of implementation, we were able to increase our residency class size by 2 residents. This allowed us to implement a required rotation while retaining 8 clinical shifts per month. In addition to allowing appropriate department coverage, we thought that retaining some clinical shifts would offer opportunities for residents to implement learning in a more typical clinical setting.

For the required rotation, we retained all 4 components of the elective (See Appendix Calendar). Based on feedback from the elective residents, we adjusted those components and added 2 more elements: a required case-based didactic presentation at our monthly case conference and one hour of PGY-1 breakout group facilitation focusing on fundamentals of emergency medicine. The residents were provided with elements from the Foundations of Emergency Medicine curriculum (https://foundationsem.com/) based on pre-selected topics. The focus of these educational sessions was to foster an engaging learning environment for the PGY-1’s through games and oral board style cases. Two faculty members with experience in medical education oversaw the breakout group experience. They provided individualized feedback to the MedEd residents regarding their teaching activity.

We also refined the process for the MedEd project. We identified a list of willing faculty mentors for the MedEd project. Residents were asked to identify an educational need in the residency, and with guidance from our education faculty and their mentor, design and execute a project to meet that need. They were given access to a running list of potential project ideas. At the end of their MedEd rotation, the senior residents presented the completed project at our monthly education faculty meeting and received feedback. Senior residents showcased their completed MedEd projects semi-annually to the entire residency program. Most projects fell under the cognitive or technical domains. Examples included infographic posters, videos simplifying concepts and simulation trainers. While not required or specifically intended as such, several residents were able to publish their MedEd projects. Example projects can be viewed here: https://sites.google.com/view/lluem/meded-rotation/meded-projects.

Our final list of required readings included various chapters from Rogers’ *Practical Teaching in Emergency Medicine*,^10^ Rogers’ *The Seven Habits of Highly Effective Medical Educators*,^11^ Green’s *Top 10 Ideas to Improve Your Bedside Teaching in a Busy Emergency Department*,^12^ and Wolff’s Not *Another Boring Lecture: Engaging Learners with Active Learning Techniques*.^13^ We taught our residents several specific teaching techniques through additional reading and discussion, including the one minute preceptor,^14^ SNAPPS,^15^ teaching scripts^16^ and bedside presentations.^17^

Our Institutional Review Board reviewed this project and determined that it did not meet the definition of human subjects research. At the end of the year, we asked each resident to complete a survey with 5-point Likert scale and free response questions. Thirteen out of fourteen senior residents completed the survey. Ten residents (77%) felt that the rotation was “moderately” or “quite” relevant to their training. Five residents (38%) reported that the rotation had “some” or more impact on their career decision. The other 8 residents reported “almost no impact” or “a little bit of impact.” Ten residents (77%) reported that they would “sometimes,” “often,” or “almost always” use the teaching techniques they learned during the rotation. The most highly rated activities were the Acute Care Skills Evaluations and teaching shifts with medical students. The lowest rated activities were the assigned regular ED shifts and the assigned readings. The most commonly cited rotation strength was the ability to focus on teaching at a slower pace without department flow pressure. Recurrent recommendations for improvement included additional simulation time, additional dedicated teaching shifts and fewer regular ED shifts.

We compared confidence with bedside teaching before and after the rotation using a Wilcoxon matched-pairs test. Confidence improved after the session, with a median confidence before the session of 3/5 (moderately confident; IQR 2–3) and a median confidence after the session of 4/5 (quite confident; IQR 3–4, p=0.006).

### Evaluation and Feedback

Although it was not our first choice, we are grateful that we deployed the rotation as an elective prior to initiating the required rotation. We recommend this step to programs considering a MedEd rotation. It allowed us to fine tune the curriculum and adjust logistics prior to deploying the month as a required rotation. We also recommend that programs considering a MedEd rotation have formal, regular medical student teaching activities in place that residents can assimilate into, especially related to simulation and direct medical student bedside teaching. These activities provided supervising faculty with opportunities to directly observe residents’ teaching and provide feedback. We suspect this is the explanation for residents’ high ratings of these activities.

The career impact of the rotation was lower than we hoped. This may be related to the rotation occurring in the third year, when many residents have already made career decisions. This was likely especially true for residents rotating during the end of the year. Currently, our residents choose their block rotation through a lottery, so while it is possible for residents considering an academic career to choose a block plan with an early MedEd rotation, we would prefer to expose all residents to teaching and academic activities earlier. Moving the rotation to the second year would allow for more time to impact career decisions.

In the future, we plan to further incorporate medical student clerkship activities, especially related to hands-on teaching. We will look to MedEd residents to expand our current skills training curriculum by leading the development of new modules. We will also explore opportunities for residents to gain teaching experience with advanced practice providers and nursing staff. We would like to eventually expand the length and number of dedicated teaching shifts during the month, replacing the standard shifts with true “junior faculty” shifts and the additional activities with students and staff.

### Appendices

- Calendar

## Figures and Tables

**Table t2-jetem-6-3-c1:** 

Topic	Recommended Educational Strategy	Educational Content	Objectives	Learners	Timing, Resources Needed (Space, Instructors, Equipment, Citations of JETem pubs or other literature)	Recommended Assessment, Milestones Addressed
Teaching medical students	Small group shift	-Post graduate year 3 (PGY-3) resident serves as a junior faculty to 4 medical students who are rotating in the Emergency Department (ED). -Bedside teaching based on patient presentation -Didactic teaching -Provide feedback -Supervise procedures	-Identify learners by level of training and progression of skill and knowledge -Learn and refine bedside teaching techniques -Develop skill in learner evaluation and feedback delivery	Fourth year medical students	5-hour shifts Instructors: 1 resident and 1 faculty member Learners: 4 medical students	Assessment: Direct observation and evaluation by the faculty member Evaluation by medical students
Teaching junior residents	Post graduate year 1 (PGY-1) breakout group	PGY-3 resident engages PGY-1 residents in a small group setting to learn about bread-and-butter topics in emergency medicine	-Identify learners by level of training and progression of skill and knowledge -Facilitate small group learning sessions	PGY-1 residents	1 hour	Assessment: Evaluations of breakout group activities
Didactics	Case Conference presentation	PGY-3 resident delivers teaching pearls and tips/tricks from clinical practice	-Identify learners by level of training and progression of skill and knowledge	Medical students, PGY-1, PGY-2, and PGY-3 residents	30 minutes	Assessment: Lecture evaluations by attendees
Simulation	Acute Care Skills Evaluation (ACSE)	PGY-3 resident runs simulation cases related to resuscitation with medical students	-Lead simulation-based learning sessions -Develop skill in learner evaluation and feedback delivery	Fourth year medical students	7–8 hours, bimonthly	Assessment: Direct observation and evaluation by faculty member
Medical Education (MedEd) area of interest	MedEd project	PGY-3 resident identifies an educational need in the residency, and with guidance from our education faculty and the mentor, designs and executes a project to meet that need	-Produce a high quality MedEd project	Medical students, PGY-1, PGY-2, and PGY-3 residents	Throughout the rotation	Assessment: Evaluation by faculty educators in the ED

**Figure f1-jetem-6-3-c1:**
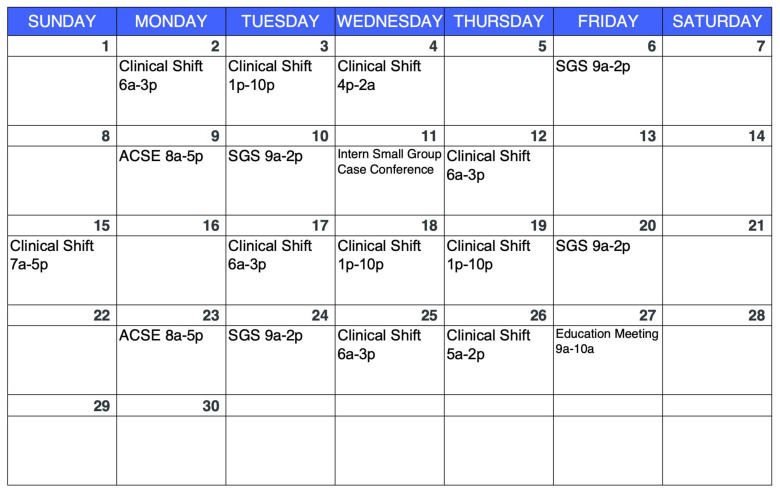
Calendar showing a typical MedEd rotation month. SGS: Small Groups Shift (medical student teaching shift); ACSE: Acute Care Skills Evaluation (medical student simulation teaching)

## DIDACTICS AND HANDS-ON CURRICULUM

[Table t2-jetem-6-3-c1]

## Appendix A Calendar

[Fig f1-jetem-6-3-c1]
